# Lignans and Other Constituents from Aerial Parts of *Haplophyllum Villosum*

**DOI:** 10.3390/molecules16032268

**Published:** 2011-03-07

**Authors:** Parimah Parhoodeh, Mawardi Rahmani, Najihah Mohd Hashim, Mohd Aspollah Sukari, Gwendoline Ee Cheng Lian

**Affiliations:** Department of Chemistry, Faculty of Science, Universiti Putra Malaysia, 43400 Serdang, Selangor, Malaysia

**Keywords:** *Haplophyllum villosum*, Rutaceae, eudesmin A, Eudesmin, haplamine

## Abstract

During our phytochemical investigation of *Haplophyllum villosum* (Rutaceae), a perennial herb from Iran, a new 4,8-diaryl-3,7-dioxobicyclo-(3,3,0)-octane type lignan, eudesmin A (**1**), together with four known compounds–eudesmin (**2**), haplamine (**3**), umbelliferone (**4**) and scopoletin (**5**)–were isolated from aerial parts of the plant. The structures of the compounds were elucidated using NMR spectral analysis (^1^H-NMR,^13^C-NMR, HSQC, COSY and HMBC) as well as UV, IR and MS spectra and comparison with previously reported data.

## 1. Introduction

**Figure 1:**
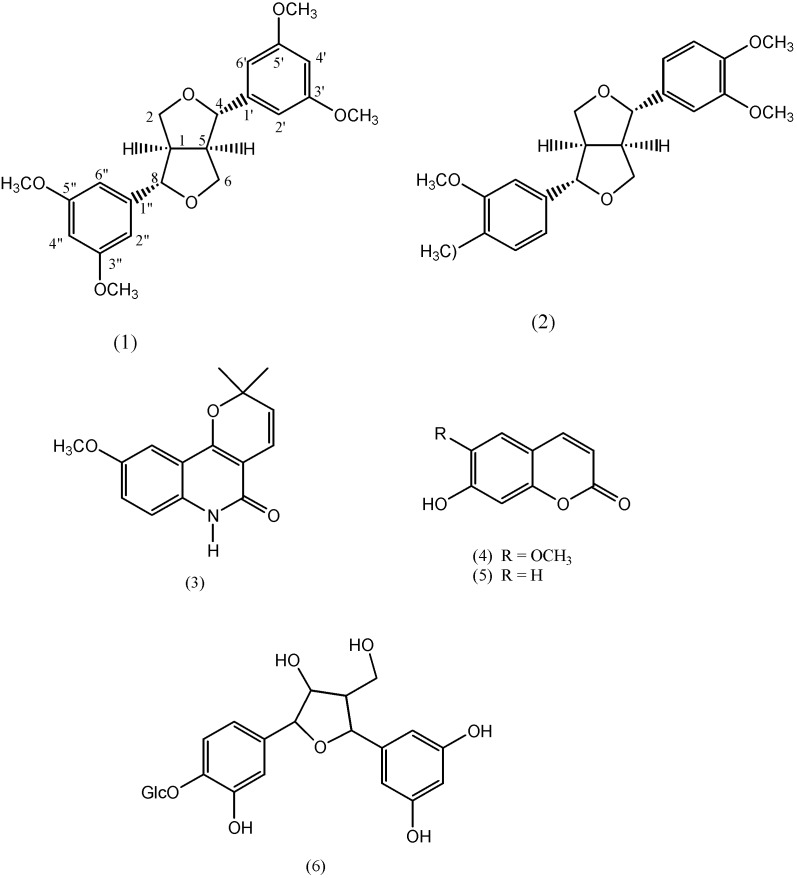
Compounds identified from *H. villosum*.

The genus *Haplophyllum* of the family Rutaceae is made up of about 70 species distributed worldwide, with about 30 of them being found in Iran. They are mainly found around the Mediterranean region of Europe and through western Asia up to Siberia [1]. Various members of the genus are used in traditional medicine for the treatment of herpes, warts, erysipelas, toothache, stomach, skin diseases [2] and in the treatment of testicular cancer [3]. The use of *Haplophyllum villosum* in Saudi Arabia to heal gynecological disorders, malaria and rheumatoid arthritis has also been reported [4]. Previous works on *Haplophyllum* species have revealed the presence of a number of aromatic compounds such as lignans, coumarins and various classes of alkaloids [5,6,7,8,9]. In continuation of our work on bioactive phytochemicals from Rutaceous plants, we wish to report the identification of a new lignan named eudesmin A (**1**, Figure 1), isolated from *H. villosum* together with four other known compounds: eudesmin (**2**), haplamine (**3**), umbelliferone (**4**) and scopoletin (**5**).

## 2. Results and Discussion

Compound **1** was obtained from the MeOH extract of the aerial plant parts as colorless needles with m.p. 92–94 °C. Its IR spectrum exhibited absorption bands at 1,514 and 1,261–1,220 cm^−1^ for the presence of an aromatic nucleus and C–O–C functionality, respectively. The EIMS gave a molecular ion peak at *m/z* 386, while the HREIMS gave *m/z* 386.1729 which correspond to a molecular formula of C_22_H_26_O_6_ (calculated 386.1744). The integration of the ^1^H-NMR spectrum showed 13 protons, half of the total number of protons in the molecular formula as calculated from the mass spectrum. This clearly indicated the presence of symmetry elements in the molecule. This was further supported by the ^13^C-NMR spectrum with the observation of ten carbon resonances in which one of the signals was an overlapped one for two carbons. These resonances were due to the presence of one methylene, two methoxyls, five methines and three quartenary carbon atoms, as could be seen in the DEPT spectra. Two sharp singlets at δ 3.80 and 3.83 each integrating for three protons were noted in the ^1^H-NMR spectrum and assigned to the two methoxyl groups at positions C-3’/3” and C-5’/5”.

The IR spectrum did not indicate the presence of any hydroxyl or carbonyl functionalities, hence the oxygen atoms were assigned to ether linkages. In view of the known occurrence of lignans in *Haplophyllum* species, this compound was tentatively identified as a 4,8-diaryl-3,7-dioxobicyclo-(3,3,0)-octane type lignan. In the aromatic region, three protons were observed with the occurrence of a broad singlet at δ 6.91 that integrated for two protons due to the two equivalent protons at H-2’/H-2” and H-6’/H-6”. Another broad singlet at δ 6.97 integrating for one proton is assigned to the protons at H-4’/H-4”. These two groups of protons (H-2’, H-4’, H-6’) and (H-2”, H-4”, H-6”) are all *meta*-coupled to each other and on this occasion the coupling constants could not be calculated because the signals occurred as two broad singlets (Table 1). These types of couplings indicated a 1,3,5-trisubstitution pattern of the aromatic ring. In comparison with its isomer, eudesmin (**2**), the aromatic protons occurred as an ABX system with the observations of a doublet of doublet at δ 6.94 (H-6’/6”) and two doublets at δ 7.02(H-2’/2”) and 6.92 (H-5’/5”) (Table 1) [10].

**Table 1:** ^1^H- and ^13^C-NMR data of eudesmin A (**1**) and eudesmin (**2**).

H/C	Eudesmin A (1) (CDCl_3_)			Eudesmin (2) (acetone- *d*_6_) [10]	
	δ_H_	δ_C_	HMBC	δ_H_	δ_C_
1/5	3.13 (m, 2H, H-8/8’)	53.5	C-2/6, C-4/8	3.10-3.14 (m, 2H, H-8/8’)	54.63
2/6	4.22 (dd, *J* = 9.2, 7.3 Hz, 2H, H-2b/6b)	77.0	C-1/5, C-4/8	3.85 (dd, *J* = 4.0, 11.0 Hz, 2H, H-9b/9b’)	71.67
	4.24 (dd, *J* = 9.2, 7.3 Hz, 2H, H-2a/6a)		C-1/5, C-4/8	4.25 (dd, *J* = 5.5, 8.0 Hz, 2H, H-9a/9a’)	
4/8	4.73 (d, *J* = 4.6 Hz, 2H,H-7/7’)	91.5	C-6/2, C-5/1	4.73 (d, *J* = 4.5 Hz, 2H,H-7/7’)	85.85
1’/1”	-	139.5	-	-	134.81
2’/2”	6.91 (br s, 2H, H-2/2’)	124.1	C-4’/4”,C-1’/1”	7.02 (d, *J* = 1.0 Hz, 2H,H-2/2’)	110.46
3’/3”	-	154.9	-	-	149.85
4’/4”	6.97 (br s, 2H, H-4/4’)	115.4	C-3’/3”,C-2’/2”	-	149.21
5’/5”	-	154.4	-	6.92 (d, *J* = 8.5 Hz, 2H,H-5/5’)	112.11
6’/6”	6.91 (br s, 2H, H-6/6’)	117.1	C-4’/4”,C-1’/1”	6.94 (dd, *J* = 2.0, 6.0 Hz, 2H, H-6/6’)	118.46
2xOCH_3_	3.80 (s, 6H, 5- and 5’-OCH_3_)	59.7	C-3’/3”	3.82 (s, 6H, 3- and 3’-OCH_3_)	55.56
2xOCH_3_	3.83 (s, 6H, 3- and 3’-OCH_3_)	59.7	C-5’/5”	3.84 (s, 6H, 4- and 4’-OCH_3_)	55.56

In lignans the *para*-position of the aromatic ring is normally oxygenated with hydroxyl, methoxyl or methylenedioxy groups, but it is not the case with the current compound, as can be seen in the multiplicity of the signals and their couplings. This argument is further substantiated by a recent publication of a new 9-norlignan glycoside **6** (Figure 1) from *Cestrum diurnum* L. in which one of the aromatic rings is also unoxygenated at the *para*-position and the three aromatic protons occurred atδ 6.71 (2H) and 6.87 (1H) as two broad singlets [11]. The confirmation of the suggested structure was established by the HMBC spectrum which demonstrated ^2^*J* and ^3^*J* multiple bond ^1^H-^13^C correlations in the molecule. The broad singlet at the aromatic region δ 6.91 clearly showed correlations to δ 115.4 (C-4’/4”) and 139.5 (C-1’/1”). Similarly, long range correlations could also be seen between the broad singlet at δ 6.97 (H-4’/4”) to 154.4 (C3’/3”) and 124.1 (C-6’/6”). Moreover, ^3^*J* correlations between methoxyl protons at δ 3.80 and 3.83 to δ 154.4 (3’/3”) and 154.9 (C-5’/5”) were clearly displayed, confirming the attachment of the two methoxyl groups at positions C-3’/3’’ and C-5’/5” and the compound is identified as [4,8-bis(3,5-dimethoxyphenyl)-3,7-dioxabicyclo-[3,3,0]octane] and named eudesmin A (**1**).

Eudesmin (**2**) was also isolated from all three extracts as colorless prisms with m.p. 103–105 °C ([12], m.p. 103–104 °C). The molecular formula is identical to that of compound **1**, with a molecular ion peak noted at *m/z* 386. Detailed structural assignment of **2** was made based on various spectral analyses and comparison with reported data for the compound isolated from *Magnolia kobus* DC. var. *borealis* Sarg [10]. Similarly, the quinoline alkaloid haplamine (**3**) was also isolated from all three extracts as colorless needles with m.p. 200–201 °C ([13], m.p. 201–202 °C). This is the only alkaloid isolated from the plant. The EIMS gave a molecular ion peak at *m/z* 257 which corresponds to the molecular formula C_15_H_15_NO_3_). The NMR spectral data of the compound are identical to those reported for haplamine by Campbell *et al.* [14]. The last two compounds, **4** and **5**, are simple coumarins and spectral analysis indicated that both were identical to umbelliferone and scopoletin, previously identified from the root of *Dystaenia takeshimana* [15] and *Haplophyllum patavinum* [16], respectively. All five isolated compounds are new to this particular plant. Both the CHCl_3_ and MeOH extracts exhibited strong antioxidant activity when tested against free radical scavenging effect of DPPH with IC_50_ values of 220 and 61 μg/mL, respectively. However, none of the isolated compounds gave any positive results.

## 3. Experimental

### 3.1. General

All melting points (m.p.) were determined using a Leica hot stage melting point apparatus model GALEN III equipped with microscope and are uncorrected. UV and IR spectra were measured with Shimadzu UV 2100 and Perkin Elmer FTIR (model 1725X) spectrophotometers, respectively. The^1^H-NMR and ^13^C-NMR spectra were obtained with JEOL ECA-400 spectrometer operating at 400 and 100 MHz with tetramethylsilane (TMS) as internal standard, respectively. The MS were obtained with a Shimadzu GCMS-QP5050 spectrometer with Direct Induction Probe (DIP) using ionization induced by electron impact at 70 eV. Column chromatography was packed with Merck silica gel 60(0.063–0.200 mm mesh size). Analytical thin layer chromatography (TLC) was performed on commercially available Merck TLC plastic sheets pre-coated with Kieselgel 60 F_254_, 0.2 mm thickness and the chromatotron plates were coated with Keisegel 60 PF_254_ and scraped to 0.75 mm thickness.

### 3.2. Plant Material, Extraction and Isolation

The aerial parts of *Haplophyllum villosum* were collected in West Azerbaijan Province, Iran in May 2008. A voucher specimen for each plant was deposited in the Shiraz University herbarium (Shiraz, Iran). The collected plant was dried under shade and ground to fine powder (1.5 kg). The material was extracted sequentially with redistilled hexane, chloroform and methanol. The solvents were removed by rotary evaporator to give 19.5, 26.5 and 93 g of dark viscous extracts, respectively. Each extract was repeatedly chromatographed over silica gel and eluted with various mixtures of hexane, CHCl_3_, EtOAC and MeOH of increasing polarity. Chromatographic separation of the hexane extract yielded eudesmin (**2**, 54 mg) and haplamine (**3**, 41 mg). Another batch of eudesmin (**2**, 50 mg) and haplamine (**3**, 28 mg) together with umbelliferone (**4**, 108 mg) were obtained from the CHCl_3_ extract, while the methanol extract again furnished eudesmin (**2**, 29 mg) and haplamine (**3**, 10 mg) together with scopoletin (**5**, 22 mg) and the new compound eudesmin A (**1**, 20 mg).

### 3.3. Spectral Data

*Eudesmin A* (**1**). Colorless needle-shaped crystals with m.p. 92–94 °C. The compound gave a dark red spot on TLC plate when viewed under short wave UV light (254 nm) developed with a solvent system of 40% EtOAc:60% hexane, and a R_f_ value of 0.31. UV λ_max_ nm (log ε), CHCl_3_: 281.1 (1.36) 242.1 (1.88). IR ν_max_ cm^−1^ (KBr disc): 3,018, 1,514, 1,261, 1,220, 1,027 and 750. EIMS *m/z* (% rel. int.): 386 (65), 355 (9), 219 (32), 205 (10), 189 (20), 177 (78), 165 (100), 151 (82), 138 (34). HREIMS *m/z*: 386.1744 [M]^+^ (calcd for C_22_H_26_O_6_, 386.1740). (^1^H-NMR (400 MHz, CDCl_3_): see Table 1. ^13^C-NMR (100 MHz, CDCl_3_): see Table 1.

*Eudesmine* (**2**). Colorless prisms with m.p. 103–105 °C ([12], m.p. 103–104 °C) and R_f_ value of 3.31 (40% EtOAc:60% Hexane). UV λ_max_ nm (log ε), CHCl_3_: 281.1 (1.44) and 243.3 (1.94). IR ν_max_ cm^−1^ (KBr disc): 2935, 2837, 1592, 1512, 1023, 748. EIMS *m/z* (% rel. int): 386 [M]^+^ (85), 355 (12), 219(22), 205 (10), 189 (28), 177 (68), 165 (100), 151 (72), 138 (35). ^1^H-NMR (CDCl_3_) and ^13^C-NMR (CDCl_3_): see Table 1 [10].

*Haplamine* (**3**). The compound was recrystallized from CHCl_3_ to afford colorless needle-shaped crystals with a R_f_ value of 0.24 (98% CHCl_3_:2% MeOH) and m.p. 200–201 °C ([13], m.p. 201–202 °C). The UV, IR, MS, ^1^H-NMR and ^13^C-NMR spectral data were identical to published data [14].

*Umbelliferone* (**4**). Obtained as colorless needle-shaped crystals with m.p. 223–225 °C ([15], m.p. 224–225 °C) after recrystallization from chloroform. The UV, IR, MS, ^1^H-NMR and ^13^C-NMR spectral data are identical to published data [15]. 

*Scopoletin* (**5**). Showed a blue fluorescent spot on TLC plate when visualized under long wave UV light developed with EtOAc:hexane (1:4) with a R_f_ value of 0.12. m.p and m.p. 200–203 °C ([16], m.p.202–204 °C). The UV, IR, MS, ^1^H-NMR and ^13^C-NMR spectral data are identical to published data [16].

## 4. Conclusions

A new lignan identified as [4,8-*bis*(3,5-dimethoxyphenyl)-3,7-dioxabicyclo[3,3,0]octane] (**1**) to which the trivial name eudesmin A has been assigned was isolated and identified from the aerial extracts of *Haplophyllum villosum* along with another lignan, a quinoline alkaloid and two coumarins. The crude extracts were found to be good free radical scavenger but none of the compounds exhibited such behaviour.
